# Liver transcriptome analysis of Atlantic cod (*Gadus morhua*) exposed to PCB 153 indicates effects on cell cycle regulation and lipid metabolism

**DOI:** 10.1186/1471-2164-15-481

**Published:** 2014-06-17

**Authors:** Fekadu Yadetie, Odd André Karlsen, Marta Eide, Christer Hogstrand, Anders Goksøyr

**Affiliations:** Department of Molecular Biology, University of Bergen, Bergen, Norway; Department of Biology, University of Bergen, Bergen, Norway; Diabetes and Nutritional Sciences Division, King’s College London, London, UK

## Abstract

**Background:**

Polychlorinated biphenyls (PCBs) are persistent organic pollutants (POPs) with harmful effects in animals and humans. Although PCB 153 is one of the most abundant among PCBs detected in animal tissues, its mechanism of toxicity is not well understood. Only few studies have been conducted to explore genes and pathways affected by PCB 153 by using high throughput transcriptomics approaches. To obtain better insights into toxicity mechanisms, we treated juvenile Atlantic cod (*Gadus morhua*) with PCB 153 (0.5, 2 and 8 mg/kg body weight) for 2 weeks and performed gene expression analysis in the liver using oligonucleotide arrays.

**Results:**

Whole-genome gene expression analysis detected about 160 differentially regulated genes. Functional enrichment, interactome, network and gene set enrichment analysis of the differentially regulated genes suggested that pathways associated with cell cycle, lipid metabolism, immune response, apoptosis and stress response were among the top significantly enriched. Particularly, genes coding for proteins in DNA replication/cell cycle pathways and enzymes of lipid biosynthesis were up-regulated suggesting increased cell proliferation and lipogenesis, respectively.

**Conclusions:**

PCB 153 appears to activate cell proliferation and lipogenic genes in cod liver. Transcriptional up-regulation of marker genes for lipid biosynthesis resembles lipogenic effects previously reported for persistent organic pollutants (POPs) and other environmental chemicals. Our results provide new insights into mechanisms of PCB 153 induced toxicity.

**Electronic supplementary material:**

The online version of this article (doi:10.1186/1471-2164-15-481) contains supplementary material, which is available to authorized users.

## Background

Polychlorinated biphenyls (PCBs) are a class of compounds composed of 209 congeners previously used as industrial chemicals, for example in electrical equipment as flame retardants, in paints, pesticides, lubricants and sealants [1]. Although PCBs were banned in the late 1970s in the Western world, they are still one of the most problematic environmental contaminants due to their extreme persistence in the environment [2]. Because of their lipophilic nature and resistance to metabolic degradation, PCBs bioaccumulate in animals and biomagnify in the food chain [1]. Consumption of fish from contaminated areas is one of the main sources of exposure to humans [3]. Based on chemical structure, PCBs are classified into two major groups with different modes of toxicity, i.e. the dioxin-like co-planar PCBs that bind the aryl hydrocarbon receptor (AhR) (e.g. PCB 126) and the non-coplanar PCBs such as PCB 153 [1, 4]. The co-planar PCBs have toxicity mechanisms mainly mediated by AhR, with a range of toxic effects including carcinogenicity, developmental toxicity, neurotoxicity, immune suppression and endocrine disruption [1]. The non-coplanar PCBs such as PCB 153 do not bind to and activate AhR, and their mechanisms of toxicity are at present less well understood. The non-coplanar PCBs such as PCB 153 may act via the steroid and xenobiotic receptor (SXR), also known as pregnane X receptor (PXR), and the constitutive androstane receptor (CAR) [5, 6].

PCB 153 is one of the PCB congeners often detectable in biological samples [1, 4], and it is commonly used as a representative compound for the non-dioxin-like (non-coplanar) PCB congeners in toxicological investigations. It is among the most persistent congeners, with residence time in the environment exceeding 100 years [2]. Toxic effects of PCB 153 include possible endocrine disruption [7, 8], neurotoxicity [9], and liver tumor promotion [10]. Accumulating evidences suggest that PCBs and other persistent organic pollutants (POPs) may also act as metabolic disruptors contributing to growing incidence of metabolic diseases [11–17]. A recent study showed that PCB 153 exacerbated obesity in mice when administered in combination with a high-fat-diet resulting in increased visceral adiposity, hepatic steatosis, plasma adipokines, as well as up-regulation and down-regulation of genes for hepatic lipid biosynthesis and degradation, respectively [18]. In mouse fibroblast 3T3-L1 cells, PCB 153 and other environmental chemicals have been shown to promote adipogenesis [16, 19, 20]. Studies using zebrafish (*Danio rerio*) suggest that fish can be useful models in exploring effects of environmental chemicals that may act as metabolic disruptors [21–23]. To date, no studies have explored effects PCB 153 toxicity in fish liver using high throughput approaches, perhaps partly due to lack of sequenced and annotated genomes for many fish species. The Atlantic cod is a commercially important species that is also commonly used in monitoring environmental pollution and laboratory toxicological investigations [9, 24–26]. The sequencing of its genome [27] has recently enabled us to apply genomic approaches to study toxicant effects in this organism [25, 28]. Exposure to individual PCB congeners, such as PCB 153, and subsequent analysis using global approaches like transcriptome assays should enable a better understanding of the toxic effects and the mechanisms involved. Such mechanistic toxicogenomics studies are increasingly recognized as important components in developing models that can help in risk assessment of environmental contaminants [29–31]. Furthermore, high throughput transcriptomics approaches may facilitate identification of new biomarkers that can be applied in improved monitoring of pollution in the aquatic environment.

The aim of this study was to map the range of molecular targets of PCB 153 and gain better insights into its toxicity mechanisms in the liver of Atlantic cod, using recently developed oligonucleotide arrays [28]. A genome-wide transcriptome analysis of Atlantic cod liver was performed after exposure to PCB 153, and various bioinformatics approaches were applied to explore the major genes and pathways affected, and the possible toxicity mechanisms involved.

## Results and discussion

### Differentially regulated genes

**Figure 1:**
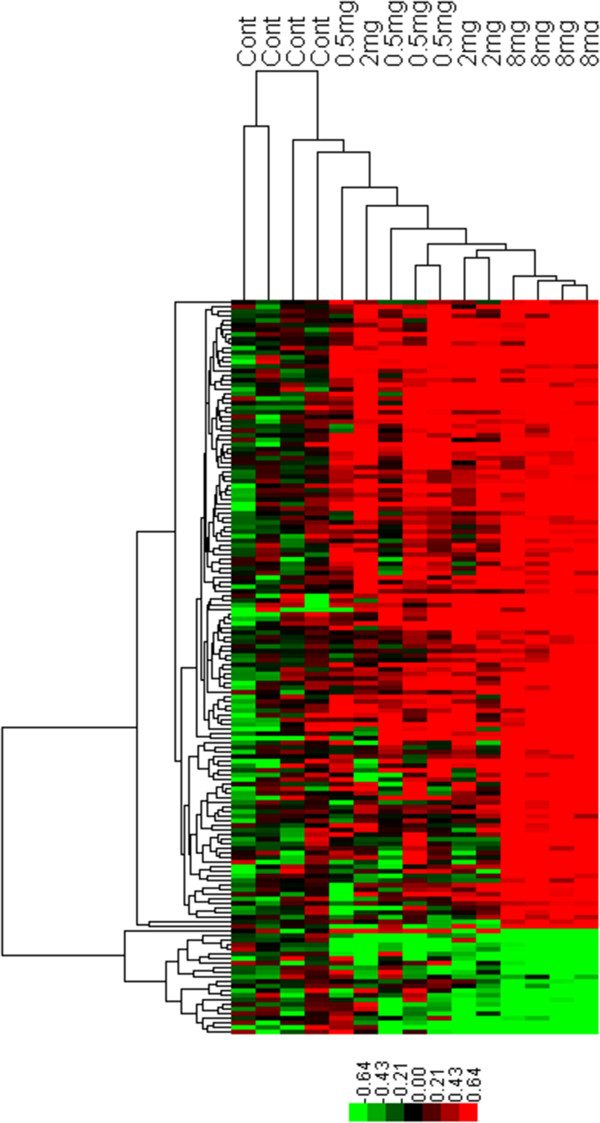
**Hierarchical clustering analysis of genes differentially regulated by PCB 153.** Analysis was performed based on log_2_-transformed ratio values of 160 genes differentially regulated, between the 8 m/kg BW PCB 153 dose and control groups. Rows represent genes and columns represent samples. Samples: Cont, Control; 0.5 mg, 2 mg and 8 mg indicate, 0.5, 2 and 8 mg/kg BW PCB 153, respectively. Color bar indicates log_2_-transformed ratio values and corresponding colors (red, black and green for up-regulated, not changing and down regulated, respectively).

Analysis of the microarray data resulted in 160 candidate genes differentially regulated in the highest dose (8 mg/kg BW PCB 153) group, using SAM (Significance Analysis of Microarrays) at FDR (False Discovery Rate)  10% (Additional file 1:Table S1). Five genes found to be differentially regulated by qPCR (Quantitative real-time polymerase chain reaction) assay (see below) were also included in Additional file 1: Table S1. This list of 165 differentially regulated genes was used for pathway enrichment analysis in DAVID (Database for Annotation, Visualization and Integrated Discovery) and MetaCore (see below). Although no differentially regulated genes were detected for the lower 0.5 and 2 mg/kg BW PCB 153 doses (at the 10% FDR cut-off), a dose–response trend is apparent in the expression of the differentially regulated genes as indicated by hierarchical clustering analysis (Figure 1). In the hierarchical clustering, the PCB 153 treated samples are well separated from the control samples, and the dose groups are co- clustered except one (2 mg/kg BW) sample that was grouped with the lowest dose group. In general high individual variability was observed in the microarray data, which combined with a limited number of samples (n = 3-4), appear to have contributed to moderate levels of statistical significance for differential regulation. Thus, Gene Set Enrichment Analysis (GSEA), which is an independent approach for pathway enrichment analysis that uses the whole set of genes without pre-selection [32], was also performed in addition to pathway analysis using DAVID and MetaCore performed with the differentially regulated genes. In addition, selected genes in the affected pathways were assayed by qPCR on a larger sample size to confirm the microarray data (see below).

### qPCR assay

**Figure 2:**
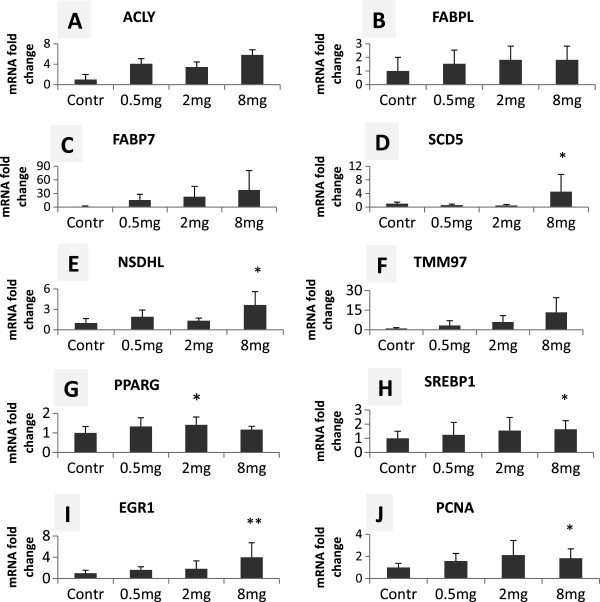
**Selected genes up**-**regulated in PCB 153 treated fish in qPCR assay.** Each panel represents a graph of fold-changes in mRNA levels for the indicated gene in lipid metabolism **(A-H)** and cell cycle **(I and J)** related pathways. qPCR was performed on a larger sample size (n = 8–10 per group) for all genes except FABP7 **(C)** for which, n = 3 for 2 mg/kg BW dose and n = 4 for each of the other groups. Contr, 0.5 mg, 2 mg and 8 mg indicate control, 0.5, 2 and 8 mg/kg BW PCB 153 doses, respectively. *p < 0.05, **p < 0.01 (one-way ANOVA and Dunnett’s multiple comparison post-test).Data are presented as mean ± standard deviation.

To confirm the microarray results in a larger number of samples (n = 8–10 per group), the mRNA levels of 10 up-regulated genes selected based on their relevance in the most enriched lipid metabolism and cell cycle related pathways were analyzed by qPCR (Figure 2). Half of these genes were selected from the differentially regulated list (Additional file 1: Table S1). Another five genes (*FABP7*, *TMM97*, *PPARG*, *SREBP1* and *PCNA*) that were up-regulated by microarray but not at significant levels (FDR >10) were also included in the PCR assay. A dose–response trend was observed for all the genes and statistical analysis using one-way ANOVA showed 6 of the 10 genes were significantly up-regulated in at least one dose group of PCB 153 treated fish as determined by qPCR assays (Figures 2A-J), thus confirming and strengthening the microarray results.

Validation of the microarray method by qPCR was also performed by direct comparison of fold-changes obtained by the two methods only in the subset of samples analyzed by microarrays (n = 3–4 per group). Expression levels of 7 of the above genes and additional randomly selected 5 genes (*MCM5*, *ADK*, *APOH*, MTL2A and *SRSF1*) from the differentially regulated list (Additional file 1: Table S1) were analyzed. For most of the genes, fold-changes in expression levels with the two methods showed good correlations (Additional file 2: Figures S1A-J). Only two of the 12 genes (*SRSF1* and *APOH*) showed poor correlations with microarray data (Additional file 2: Figures S1K and L). Confirming the microarray data, 10 out of the 12 genes were significantly up-regulated (*p* < 0.05, one-tailed Student’s t-test) in the highest dose group (Additional file 2: Figures S1A-J). Thus, in general there is good concordance between the two methods.

### Functional enrichment analysis

### Pathway and gene ontology analysis in DAVID

**Figure 3:**
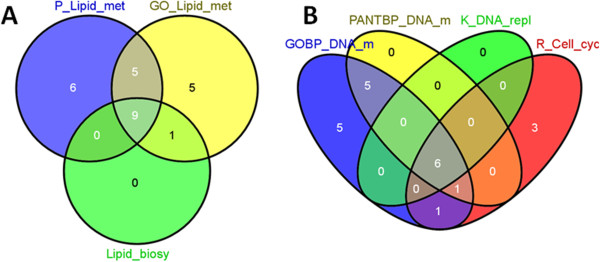
**Venn diagrams showing overlapping genes in enriched lipid metabolism (A) and DNA metabolism**/ **cell cycle (B) pathways and processes in Table** 1. Abbreviations for terms in Table 1: P_Lipid_met, PANTHER_BP Lipid, fatty acid and steroid metabolism; GO_Lipid_met, GOTERM_BP Lipid metabolic process; Lipid_biosy, GOTERM_BP Lipid biosynthetic process. GO_DNA_met, GOTERM_BP DNA metabolic process; GO_DNA_repl, GOTERM_BP DNA replication; Resp_Stim, GOTERM_BP Cellular response to stimulus; R_Cell_cy, Reactome_Pathway, Cell Cycle, Mitotic.

The 165 differentially regulated genes (Additional file 1: Table S1) were used in pathway analysis using DAVID [33] to see to most enriched pathways and biological processes. Functional annotation in DAVID showed that two of the top three significantly enriched clusters of pathways and Gene Ontology (GO) biological processes (BP) are related to lipid metabolism and DNA replication/ cell cycle (Table 1). The different significantly enriched GO BP and pathways in each of these two major enriched pathways have overlapping list of constituent genes as illustrated by the Venn diagrams for lipid metabolism (Figure 3A) and DNA metabolism related genes (Figure 3B). For example, all or the majority of the genes in DNA Replication, DNA repair and cell cycle pathways and processes are the subset of genes in the DNA metabolism biological process (Table 1, Figure 3B). As expected, Reactome DNA replication genes are a subset of Cell cycle genes, and all the genes in GO BP Cellular response to stress are a subset of the genes in Cellular response to stimulus (Table 1). The BP Cellular response to stimulus shares many of the genes (10 of 18) with DNA metabolism and Cell cycle, suggesting that the stress response activated here is related to DNA replication/ cell cycle (Figure 3B). The last significantly enriched term (FDR < 1.3) in the last cluster contains 3 genes (*KIT*, *BAX*, and *SCRIB*) and is related to programmed cell death (not shown). In summary, the significantly enriched functional annotations suggest that pathways related to lipid metabolism and cell cycle were affected by PCB 153 in cod liver. The up-regulation of many DNA replication and mitotic cell cycle genes suggests proliferative effect of PCB 153. Similarly, up-regulation of all the genes in the Lipid metabolic process (such as *ACACA* and *ACSA*) (Table 1) suggests lipogenic effect of PCB 153.

**Table 1:** **Annotation clusters with significantly enriched GO biological processes and pathways in PCB 153 treated samples** ^**a**^

Category	Term	FDR	Gene/protein symbols
Annotation Cluster 1	Enrichment Score: 3.7		
PANTHER_BP	Lipid, fatty acid and steroid metabolism	0	ACSA, AUHM, PQLC3, SCD5, HMDH, GNPAT, PCY2, UD11, STAR3, NSDHL, ACACA, SRBP1, FABP7N, ANXA4, PCTL, GDPD2, ACLY, PPARG, FABPL, DHB12
GOTERM_BP	Lipid metabolic process	0.2	ACSA, SCD5, APOH, HMDH, GNPAT, PCY2, KIT, UD11, GPAT3, STAR3, NSDHL, ACACA, SRBP1, BAX, PLB1, GDPD2, ACLY, MK14, PPARG, DHB12
GOTERM_BP	Lipid biosynthetic process	4.2	ACSA, GPAT3, STAR3, ACACA, NSDHL, SCD5, ACLY, HMDH, PCY2, DHB12
Annotation Cluster 2	Enrichment Score: 2.2		
GOTERM_BP	DNA metabolic process	0	DNMT1, BAF, BLM, TYDP1, FEN1, NUP98, RTEL1, MCM3, PPIA, MCM5, RMI1, DPOA2, BAX, DPOD1, KC1E, FOS, PCNA, RECQ4
PANTHER_BP	DNA metabolism	0.1	MCM5, DPOA2, DNMT1, DPOD1, BLM, TYDP1, KC1E, FEN1, PCNA, RECQ4, RTEL1, MCM3
KEGG_PW	DNA replication	0.2	MCM5, DPOA, DPOD1, FEN1, PCNA, MCM3
REACT_PW	DNA Replication	0.2	MCM5, PSMD3, DPOA2, DPOD1, FEN1, PCNA, PSB7, MCM3
GOTERM_BP	DNA replication	0.5	MCM5, RMI1, DPOA2, DPOD1, BLM, FEN1, NUP98, PCNA. MCM3
PANTHER_BP	DNA repair	0.5	DPOD1, BLM, TYDP1, KC1E, FEN1, PCNA, RECQ4, RTEL1
GOTERM_BP	Cellular response to stress	0.9	SYAC, BLM, E2AK2, TYDP1, FEN1, RTEL1, ETV5, SRBP1, BAX, DPOD1, KC1E, MK14, FOS, PCNA, RECQ4
PANTHER_BP	DNA replication	1.1	MCM5, DPOA2, DPOD1, BLM, FEN1, PCNA, MCM3
GOTERM_BP	Cellular response to stimulus	1.8	SYAC, BLM, E2AK2, TYDP1, FEN1, RTEL1, ETV5, UD11, SRBP1, BAX, DPOD1, KC1E, ERBB3, MK14, PPARG, FOS, PCNA, RECQ4
GOTERM_BP	DNA-dependent DNA replication	3.0	MCM5, DPOD1, BLM, FEN1, MCM3
REACT_PW	Cell Cycle, Mitotic	4.5	MCM5, PSMD3, DPOA2, DPOD1, KC1E, KNTC1, FEN1, NUP98, PCNA, PSB7, MCM3
Annotation Cluster 3	Enrichment Score: 2.0		
GOTERM_BP	Macromolecule localization	2.4	APOH, GNPTA, SNX12, KPCB, NUP98, SNX25, DVL1L, VPS53, STAR3, EZRI, SNX18, DPOA2, BAX, YIF1A, GOT1B, DUS16, APOM, PPARG, RFIP2, PCNA, FABL

^a^Enrichment analysis was performed for functional categories GO BP (PANTHER_BP_ALL and GOTERM_BP_ALL) and pathway (KEGG and Reactome) using DAVID tools (functional annotation cluster). Only significant annotation terms (FDR < 5%) are shown. All the genes were up-regulated except four (*KIT*, *UD11*, *PLB1*, *BAF*, *E2AK2*), which were down regulated.

### Functional ontology analysis using MetaCore

**Figure 4:**
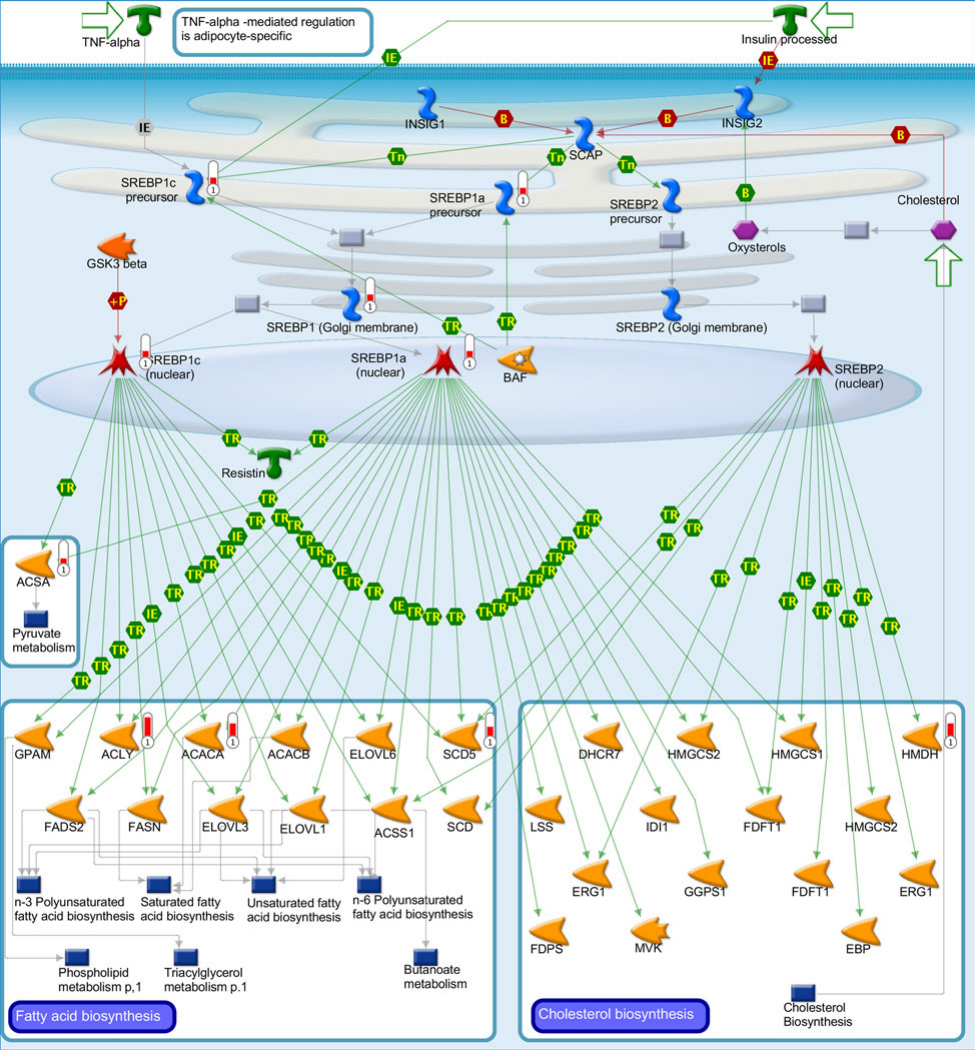
**Map of SCAP**/**SREBP Transcriptional Control of Cholesterol and FA Biosynthesis.** This is the top enriched GeneGo pathway showing the differentially regulated genes (all up-regulated) indicated by thermometer-like symbols in red. For detailed legend see Figure 2 SH in Additional file 2.

Analysis of the differentially regulated genes using the functional ontologies in MetaCore (GeneGo pathway map, process network and GO process) resulted in significant enrichment of many pathways and networks mainly related to lipid metabolism, cell cycle, tissue remodeling and wound repair, immune response, stress response, apoptosis and various signaling pathways (Table 2, Additional file 2: Tables S2-4). Larger number of processes and pathways were significantly enriched using MetaCore, perhaps because of differences in annotations between the databases used. Significantly enriched top 20 GeneGo pathways, GO processes and top 20 process networks are shown in Additional file 2: Table S2, S3 and S4, respectively. Pathways and GO processes associated with lipid metabolism and DNA metabolism are among the top significantly enriched (Additional file 2: Tables S2 and S3), which is consistent with the enrichment analysis using DAVID (Table 1). The map of the top scoring pathway related to lipid metabolism (Table 2), SCAP/SREBP Transcriptional Control of Cholesterol and FA Biosynthesis, is shown in Figure 4. All the genes indicated on this pathway including genes coding for the sterol regulatory element-binding protein 1 (SREBP1) and key genes of enzymes in fatty acid biosynthesis (e.g. ACLY, ACAC) were up-regulated (Figure 2, Additional file 1: Tables S1), suggesting increased synthesis of fatty acids. SREBP1 plays key role is transcriptional activation of the lipogenic enzyme genes in the liver [34].

**Table 2:** **Significantly enriched top 20 GeneGo pathways** ^**a**^

Maps	p-value	FDR	Gene/protein symbols
SCAP/SREBP Transcriptional Control of Cholesterol and FA Biosynthesis	6.0E-09	0	HMDH, SREBP1 (Golgi membrane), ACLY, ACSA, SCD5, SREBP1 precursor, SREBP1 (nuclear), ACACA
Regulation of lipid metabolism_Regulation of lipid metabolism via LXR, NF-Y and SREBP	2.1E-05	0	SREBP1 (Golgi membrane), ACLY, SREBP1 precursor, SREBP1 (nuclear), ACACA
Adiponectin in pathogenesis of type 2 diabetes	1.2E-04	0	SREBP1 precursor, p38alpha (MAPK14), SREBP1 (nuclear), ACACA
Immune response_Oncostatin M signaling via MAPK in mouse cells	2.6E-04	0	EGR1, PPAR-gamma, p38 MAPK, c-Fos
Development_Role of IL-8 in angiogenesis	2.8E-04	0	HMDH, SREBP1 (Golgi membrane), SREBP1 precursor, SREBP1 (nuclear), c-Fos
Immune response_Oncostatin M signaling via MAPK in human cells	3.2E-04	0	EGR1, PPAR-gamma, p38 MAPK, c-Fos
Development_Gastrin in differentiation of the gastric mucosa	3.5E-04	0	PKC-beta, EGR1, PKC, cPKC (conventional)
Development_EGFR signaling pathway	4.2E-04	0	PKC-beta, p38 MAPK, p38alpha (MAPK14), c-Fos, Bax
Regulation of lipid metabolism_Regulation of fatty acid synthase activity in hepatocytes	6.1E-04	0	SREBP1 (Golgi membrane), SREBP1 precursor, SREBP1 (nuclear)
Regulation of lipid metabolism_Insulin regulation of fatty acid methabolism	1.2E-03	0	SREBP1 (Golgi membrane), ACLY, SREBP1 precursor, SREBP1 (nuclear), ACACA
SREBP1 cross-talk with PXR, CAR and LXR	1.6E-03	0	SREBP1 (Golgi membrane), SREBP1 precursor, SREBP1 (nuclear)
G-protein signaling_Ras family GTPases in kinase cascades (schema)	1.6E-03	0	p38 MAPK, p38alpha (MAPK14), c-Fos
DNA damage_ATM / ATR regulation of G2 / M checkpoint	1.6E-03	0	BLM, p38alpha (MAPK14), GADD45 beta
Cell cycle_Transition and termination of DNA replication	2.0E-03	0.1	PCNA, FEN1, POLD cat (p125)
Apoptosis and survival_p53-dependent apoptosis	2.2E-03	0.1	p38alpha (MAPK14), GADD45 beta, Bax
Renin-Angiotensin-Aldosterone System	2.3E-03	0.1	PKC-beta, CaMK I, p38alpha (MAPK14), c-Fos
Neuroprotective action of lithium	2.4E-03	0.1	p38 MAPK, p38alpha (MAPK14), Dsh, Bax
SREBP1 cross-talk with PXR, CAR and LXR/ Rodent version	2.6E-03	0.1	SREBP1 (Golgi), SREBP1 precursor, SREBP1 (nuclear)
Development_Inhibition of angiogenesis by PEDF	2.6E-03	0.1	PPAR-gamma, p38 MAPK, Bax
DNA damage_ATM/ATR regulation of G1/S checkpoint	2.9E-03	0.1	PCNA, BLM, GADD45 beta

^a^Only the significantly enriched top 20 pathways are shown here, with the full list presented in Additional file 2: Table S2.

The organ-specific toxicity ontologies in MetaCore combine normal and pathological processes with organ-specific gene markers (GeneGo). Enrichment analysis of liver-specific toxicity ontologies was performed to explore effects on liver specific functions. Again, the top enriched liver-specific Toxic Pathology Biomarkers, Toxicity Processes, Pathway Maps, GO Processes, GO Molecular Functions, GO Localizations and Drug and Xenobiotic metabolism Enzymes highlight mainly lipid metabolism and cell proliferation related events (Additional file 2: Figures S2A-G). For example, the two top enriched Toxic Pathology Biomarkers, Liver-lipid accumulation, macrovesicular (FDR = 0.09) and Liver-degeneration (FDR = 0.13) are pathologies that might be attributed to up-regulation of lipogenic genes (Additional file 2: Figure S2A). The most significant interaction network generated in MetaCore using the 8 genes in Liver-lipid accumulation, macrovesicular is shown in (Additional file 2: Figure S2H). This network shows potential involvement of the genes in development of pathology related to lipid accumulation. Similarly, the top liver-specific Toxicity Processes, Steatosis, development (FDR = 0.15) and Cell cycle, processes involved in S-phase (FDR = 0.68) (Additional file 2: Figure S2B) are related to lipid accumulation and cell proliferation, respectively. The list of liver-specific enriched Maps and GO processes (Additional file 2: Figures S2C and D) are similar to the list in Additional file 2: Table S2 and S3, respectively. GO molecular functions and localizations also reflect the top enriched lipid metabolism and cell cycle pathways (Additional file 2: Figures S2E and F). The analysis also showed enrichment of pathways mediated by AhR and the nuclear receptors (CAR, FXR and LXR and PXR) associated with Drug and Xenobiotic metabolism Enzymes (Additional file 2: Figure S2G). In mammals, the nuclear receptors LXR, CAR and PXR are involved in regulation of lipid metabolism, mainly through cross-talk with SRBP1 pathway (See Table 2) [35]. Enrichment of PXR/ CAR mediated pathway is expected as PCB 153 is known to activate these receptors [5]. Surprisingly, AhR mediated regulation of Drug and Xenobiotic metabolism Enzymes is the top enriched pathway with 17 genes involved (Additional file 2: Figure S2G), although PCB 153 does not activate AhR. The 17 genes in this pathway are *CCL4*, *CNTNAP1*, *FOS*, *HMGCR*, *ITPKB*, *MAPK14*, *MYO1D*, *PCNA*, *POLA2*, *PSMA5*, *SREBF1* and *TEAD3*. The AhR signaling pathway may however interact with these genes indirectly and the enrichment here may reflect its diverse biological functions and possible alternative mechanisms involved.

### Interactome and network analysis using MetaCore

The MetaCore Interactome analysis tool was used to explore statistically significant interactions of the differentially regulated genes (Additional file 1: Table S1). The analysis option “Transcription Factors” was used to perform enrichment analysis of interactions based on transcriptional regulation mechanisms, which generated a list of significantly enriched (FDR < 0.05) transcription factors (Additional file 2: Table S5). Here, enrichment shows that a significantly higher proportion of genes in the up-loaded list (compared to the total number of genes in the whole database for the organism) show interactions with the transcription factor. Many transcription factors involved in regulation of the cell cycle (e. g. E2F1, E2F2, E2F3, E2F4, E2F6, EGR1and EGR2) and lipid metabolism (N220, SREBP1 and SREBP2) were significantly enriched (Additional file 2: Table S5), consistent with the top enrichment of these two pathways. Interestingly, the top enriched transcription factor N220 (ZNF638) is a regulator of adipocyte differentiation and the lipogenic transcription factors PPARG and SREBP1[36]. Among the transcription factors enriched, early growth response 1 (EGR1) and Sterol regulatory element-binding protein 1 (SREBP1) were also up-regulated in this study (Additional file 1: Table S1, Figure 2).

**Figure 5:**
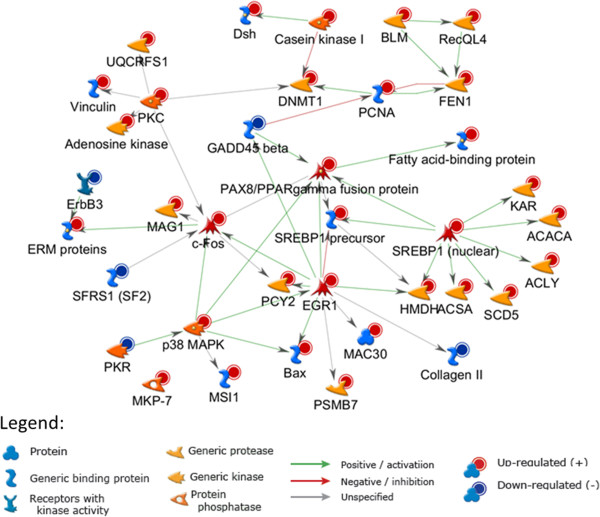
**A statistically significant network of interactions within the differentially regulated genes.** The “Direct interaction” algorithm in MetaCore was used for generation of the interaction networks. Only genes with direct connections in the network are shown.

To build a network of interaction among the differentially regulated genes in our experiment, the network building tool in MetaCore with the “Direct interaction” algorithm was used, which resulted in a statistically significant (FDR < 0.05) network (Figure 5). Remarkably, this network reveals regulatory relationships between the transcription factors and their target genes in the differentially regulated list, highlighting activation of the two main pathways lipid metabolism and cell cycle. Particularly well illustrated is the interactions of EGR1, PPARG and SRBP1 transcription factors that result in activation of the lipogenic genes *HMDH*, fatty acid binding protein, *ACACA*, *ACLY*, *SCD5* and *ACSA*, which is supported by the top enriched canonical pathway (Figure 4). The network suggests activation of the transcription factors through the MAPK (Mitogen-activated protein kinase) pathway and potential cross-talk between the cell proliferation and lipogenic pathways. The gene coding for the transcription factor EGR1 is one of the most up-regulated in this experiment (Additional file 1: Table S1, Figure 2), which may suggest its importance in the regulation of the differentially regulated genes. As indicated EGR1 appears to occupy a central role in the network as it is possibly activated via the p38 MAPK signaling pathway, and interacts downstream with both proliferation and lipogenic transcription factors (Figure 5). EGR1, known to regulate many genes related to cell proliferation, apoptosis and immune response [37, 38], has also been implicated in regulation of cholesterol biosynthesis genes [39], supporting the interactions depicted in the network shown here.

### Gene Set Enrichment Analysis (GSEA)

**Figure 6:**
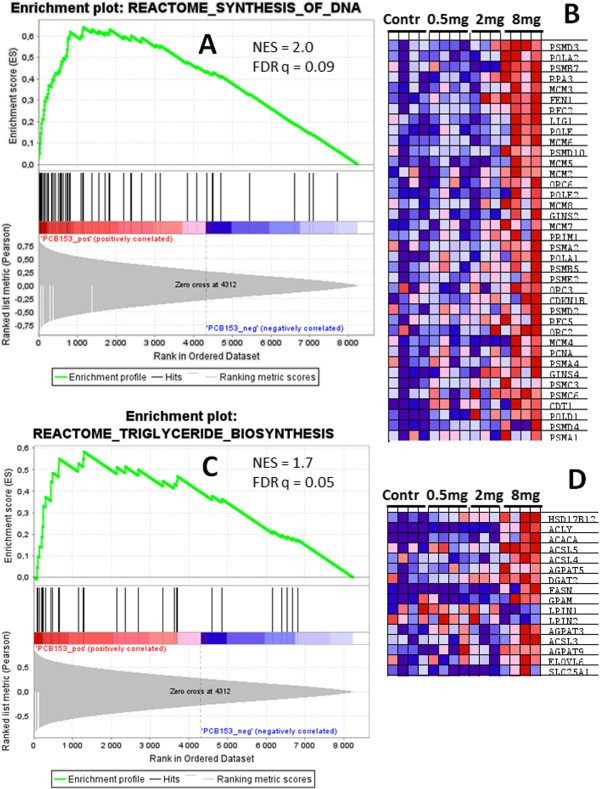
**Enrichment plots for representative gene sets in Table** 3 **.** Enrichment plots for the top genes set Reactome pathway *synthesis of DNA* **(A)** and the corresponding heat map for the “leading edge genes” **(B)**. The upper panel **(A)** shows a plot of enrichment scores (ES) *versus* rank positions of gene set members. Similar enrichment plot for the Reactome pathway *triglyceride metabolism* **(C)** and the corresponding heat map for the “leading edge genes” **(D)** are shown. On the horizontal axes **(A and C)**, the genes are sorted based on expression correlation (absolute Pearson ranking metric) with PCB 153 treated samples (genes with high correlation, top ranked on the left end). The “hit” positions (vertical lines) of genes are shown on the horizontal bars colored from deep red (top rank) to light blue (lowest rank). Genes with the hits clustered before each peak constitute “leading edge” list up- or down-regulated in PCB 153 treated samples, and are shown on the heat map on the right of each plot **(B and D)**. The heat maps show relative expression levels from deep red (highest) to dark blue (lowest) of the leading edge genes in each fish of the control, 0.5, 2 and 8 mg/kg BW PCB 153 treated groups (n = 3–4 per group) as indicated. *NES*, normalized ES; FDR *q*, False Discovery Rate q-value.

GSEA uses the whole set of genes without pre-selection to determine if a pre-defined set of genes constituting a functional category show significant, concordant differences in expression between two biological states [32]. GSEA was performed here as an independent and complementary method to the pathway analyses using DAVID and MetaCore, which were performed only on the differentially regulated genes. GSEA is suited for modest changes in gene expression since it draws its statistical power from concordant differential expression of many genes within a gene set and their correlation to one of the two biological states under comparison [40]. In this analysis, all cod genes on the array that could be mapped to putative human orthologs were ranked based on relative expression correlation from highest absolute levels in the PCB 153 groups to the lowest levels. The predominant gene sets significantly enriched are the Reactome gene sets related to DNA metabolism and cell cycle (Table 3, Additional file 3: Table S6). Table 3 shows the significantly enriched gene sets of the Reactome (only the top 20), GenMAPP (Gene Map Annotator and Pathway Profiler) and KEGG (Kyoto Encyclopedia of Genes and Genomes) databases. Gene sets related to lipid metabolism, triglyceride biosynthesis and glycerophospholipid metabolism were among the significantly enriched (Additional file 3: Table S6). Many more Reactome gene sets (total 56) were significantly enriched (FDR < 0.25), compared to only 4 and 3 for GenMAPP and KEGG, respectively (Table 3, Additional file 3: Table S6). Most of the Reactome gene sets are associated with DNA repair, replication and cell cycle (Additional file 3: Table S6). Other top enriched Reactome gene sets are mainly related to immune response, apoptosis, respiratory electron transport, translation and circadian clock. The top significantly enriched gene sets (Table 3, Additional file 3: Table S6) are largely similar to the enriched pathways using DAVID and MetaCore (Tables 1 and 2, Additional file 2: Tables S2, S3 and S4, Additional file 3: Tables S6), which mainly highlighted pathways associated with DNA metabolism, cell cycle, lipid metabolism, apoptosis, immune response and, stress response. In Figure 6, enrichment plots for top enriched Reactome pathways, synthesis of DNA (A) with the corresponding leading edge genes (B), and triglyceride biosynthesis (C), with the corresponding leading edge genes (D) are shown. Genes in the enriched gene sets have higher enrichment scores (ES) and tend to have hits clustered towards the top of the sorted gene list (Figures 6A and C), suggesting their modulation by PCB 153. The leading edge list in the top gene set synthesis of DNA (Figure 6B) contains most of the genes in the Reactome pathways DNA replication and Cell cycle enriched in pathway analysis using DAVID (Table 1). Similarly, the leading edge list in the gene set triglyceride biosynthesis (Figure 6D) consists of genes coding for enzymes in *de novo* synthesis of glycerolipids such as ACLY, ACACA, FASN, GPAT, AGPAT3, AGPAT5, and AGPAT9, some of which are also present in the enriched lipid synthesis biological processes in DAVID (Table 1). The up-regulation of these genes may indicate increased lipid synthesis in PCB 153 treated fish liver, in agreement with the results from analysis using DAVID and MetaCore.

**Table 3:** **Gene sets enriched in PCB 153 treated samples** ^**a**^

Reactome gene set	SIZE	NES	NOM p-val	FDR q-val
SYNTHESIS_OF_DNA	61	2.0	3.1E-03	0.09
DNA_STRAND_ELONGATION	25	1.9	1.4E-02	0.05
M_G1_TRANSITION	52	1.9	7.2E-03	0.04
S_PHASE	74	1.9	7.1E-03	0.05
ASSEMBLY_OF_THE_PRE_REPLICATIVE_COMPLEX	40	1.9	0	0.04
ACTIVATION_OF_THE_PRE_REPLICATIVE_COMPLEX	26	1.9	3.0E-02	0.04
ORC1_REMOVAL_FROM_CHROMATIN	41	1.8	0	0.05
ACTIVATION_OF_NF_KAPPAB_IN_B_CELLS	34	1.8	6.1E-03	0.06
SCF_BETA_TRCP_MEDIATED_DEGRADATION_OF_EMI1	25	1.8	7.2E-03	0.05
G1_S_TRANSITION	76	1.8	1.3E-02	0.05
ER_PHAGOSOME_PATHWAY	30	1.7	4.1E-03	0.06
CDT1_ASSOCIATION_WITH_THE_CDC6_ORC_ORIGIN_COMPLEX	32	1.7	3.0E-03	0.05
TRNA_AMINOACYLATION	26	1.7	2.7E-02	0.05
SCFSKP2_MEDIATED_DEGRADATION_OF_P27_P21	31	1.7	1.1E-02	0.05
MITOTIC_G1_G1_S_PHASES	90	1.7	1.6E-02	0.05
VIF_MEDIATED_DEGRADATION_OF_APOBEC3G	25	1.7	1.0E-02	0.05
TRIGLYCERIDE_BIOSYNTHESIS	30	1.7	9.1E-03	0.05
ACTIVATION_OF_ATR_IN_RESPONSE_TO_REPLICATION_STRESS	28	1.7	4.3E-02	0.06
GLOBAL_GENOMIC_NER_GG_NER	25	1.7	1.7E-02	0.06
**GenMAPP gene set**	SIZE	NES	NOM p-val	FDR q-val
DNA_REPLICATION_REACTOME	29	2.0	0.021	0.03
AMINOACYL_TRNA_BIOSYNTHESIS	15	1.9	0.020	0.03
G1_TO_S_CELL_CYCLE_REACTOME	41	1.6	0.058	0.20
GLYCEROPHOSPHOLIPID_METABOLISM	32	1.6	0.020	0.21
**KEGG gene set**	SIZE	NES	NOM p-val	FDR q-val
DNA_REPLICATION	30	1.9	0.027	0.04
NUCLEOTIDE_EXCISION_REPAIR	31	1.6	0.025	0.24
OXIDATIVE_PHOSPHORYLATION	50	1.6	0.047	0.18

^a^Only significantly enriched (FDR q-value < 0.25) top 20 Reactome gene sets and all significant GenMAPP and KEGG gene sets are shown. Gene sets are ranked by normalized enrichment score (NES). SIZE and NOM p-val, indicate number of core genes in the enriched gene set and Nominal p-value, respectively.

In summary, pathways, processes and networks related to DNA replication, cell cycle and lipid metabolism were consistently enriched using the different methods of analysis employed. The coordinated up-regulation of DNA replication and cell cycle related genes suggests that PCB 153 increases cell proliferation in the liver of cod. In agreement with our results, PCB 153 has been shown to increase hepatocyte proliferation and act as liver tumor promoter in rats [10]. Some of the up-regulated genes coding for enzymes such as ACLY, ACACA and FASN (Figure 4, Figure 6D) are important in *de novo* lipid biosynthesis pathway [41], suggesting increased lipogenesis in PCB 153 treated fish. For example, ACACA catalyzes the rate-limiting reaction in the synthesis of long-chain fatty acids [42].

Transcriptional activation of both cell cycle and lipogenic genes has been observed during adipogenesis in mammalian model systems [43]. It is not clear if the simultaneous up-regulation of cell cycle and lipogenic genes in cod liver is mechanistically related to these adipogenesis processes in mammalian systems. It is possible that up-regulation of genes associated with cell cycle progression is related to activation of immune response pathways observed here or liver tumor promoting effects of PCB 153, as shown in rats [10]. However, the transcriptional activation of lipogenic genes in cod liver treated by PCB 153 appears to be supported by recent studies that have documented adipogenic effects of PCBs and other environmental chemicals [11, 12, 15]. In mice, it was reported that bisphenol A increased hepatic expression of lipogenic genes and lipid accumulation [44]. In pre-adipogenic fibroblast 3T3-L1 cell culture studies, PCB 153 and other chemicals have also been shown to increase transcription of lipogenic marker genes, lipid accumulation and adipogenic differentiation [16, 19, 20]. A recent study showed that mice fed with a combination of PCB 153 and high-fat-diet showed increased visceral adiposity, hepatic steatosis, plasma adipokines, and up-regulation of hepatic lipid biosynthesis and down- regulation of β-oxidation genes [18]. In our experiment, significant changes were not observed in expression of β-oxidation genes, although mRNA levels of the key gene of this pathway, liver carnitine palmitoyltransferase 1A (*CPT1A*) showed a non-significant (FDR > 10%) decrease (expression ratio 0.61 in the highest dose group). Phospholipase B1 (*PLB1*) among the down-regulated genes (Additional file 1: Table S1) may also be related to lipid degradation. Our hypothesis that transcriptional activation of lipid biosynthesis genes could be attributed to lipogenic effect of PCB 153 is supported by these studies. Although species differences should be considered in the comparison with mammalian systems, our results appear to be consistent with conservation of lipid metabolism and adipogenesis processes in fish and mammals [45, 46]. Indeed, zebrafish (*D. rerio*) is increasingly used as a model organism in research on obesity and related metabolic diseases [21–23]. Thus, our results showing increased transcription of lipogenic genes in the liver of cod treated with PCB 153 can provide relevant insights into potential effects in mammals.

Apart from cell cycle and lipid metabolism pathways, other significantly enriched pathways are mainly associated with immune response, apoptosis, stress response and tissue remodeling, and they are probably related to general toxicity by PCB 153. Some of the genes in these pathways (e.g. stress response and apoptosis) have also significant overlap with the genes in lipid metabolism and cell cycle pathways (Tables 1 and 2, Additional file 2: Table S2). Apoptosis and immune response pathways have been previously reported to be affected in transcriptomics and proteomics studies in response to environmental contaminants in cod and other fish [26, 28, 47–50]. Studies in higher organisms have also shown that PCB 153 can modulate the immune system [8, 51].

## Conclusions

Gene expression analysis in the liver of cod treated with PCB 153 indicated transcriptional activation of genes mainly associated with cell cycle, DNA replication, lipid metabolism, immune response, apoptosis and stress response pathways. The constituent genes in the two major affected pathways, cell cycle and lipid biosynthesis were mostly up-regulated, suggesting increased cell proliferation and lipid synthesis in PCB 153 treated cod liver. The results provide new insights into mechanisms of toxicity of PCB 153.

## Methods

### Fish exposure and sampling

Fish exposure and sampling has been described previously [9]. Briefly, juvenile Atlantic cod of mixed genders (*G. morhua*) approximately 1.5 years old with 220–530 g of body weight (BW) were kept in 500 L tanks and supplied with continuously flowing seawater at temperature of 10°C. A 12:12 h light/dark cycle was used and the fish were fed daily for 8 h with commercial pellets (EWOS, Bergen, Norway). After acclimation for 6 days, the fish were injected (i.p.) with 0, 0.5, 2 or 8 mg/kg body weight PCB 153 (2,2′,4,4′,5,5′- hexachlorobiphenyl) (98.3% purity, Chem Service, West Chester, USA). The doses were given in two injections, half of the dose on the first day and the second half after one week. PCB 153 was dissolved in a vehicle of 20% acetone and 80% soybean oil. The control fish were injected with the vehicle only. The fish were sacrificed 14 days after the first injection and liver samples were frozen in liquid nitrogen and stored at −80°C until use. At the end of the experiment, chemical analysis showed liver burden of PCB 153 increased to approximately 4 times the maximum total body burden from the injected doses [9], reflecting the tendency of highly lipophilic compounds like PCBs to accumulate in the lipid-rich liver of the cod. The exposure experiment was approved by the National Animal Research Authority.

### RNA extraction

Total RNA isolation from liver samples, determination of concentration and quality assessment was performed as described before [28]. RNA samples from vehicle control, 0.5 and 2 and 8 mg/kg BW PCB 153 doses (n = 3 for 2 mg/kg BW dose, and n = 4 for each of the other groups) were submitted to Roche NimbleGen for labeling and hybridization.

### Microarray design and hybridization

Microarray design and hybridization has been described previously [28]. Briefly, Atlantic cod 135 K oligonucleotide arrays were designed from a 44 k cDNA collection [27] and manufactured by Roche Nimblegen (Madison, WI). The array contains 125,825 probes derived from the *G. morhua* sequences (3 or more probes per cDNA sequence) and 11,779 Nimblegen control probes. RNA samples from control fish (n = 4), 0.5 mg/kg BW PCB 153 (n = 4), 2 mg/kg BW PCB 153 (n = 3) and 8 mg/kg BW PCB 153 (n = 4) doses were submitted to Roche NimbleGen for hybridization. For each sample, double strand cDNA labelled with Cy3 was hybridized on the array according to protocols in Gene Expression User Guide (Roche NimbleGen, Madison, WI).

### Microarray data analysis

Filtering and analysis of differential regulation was performed essentially as described before [28], with the following modifications. Expression ratios were (calculated by dividing fluorescent intensity values in each sample by average intensity value of control sample), log_2_-transformed and differentially regulated genes were identified using Significance Analysis of Microarrays (SAM) [52] implemented in J-Express [53] (Molmine, Bergen, Norway). For identification of differentially regulated genes, the control and 8 mg/kg BW PCB 153 dose groups were compared using SAM, and genes were considered differentially regulated at a maximum False Discovery Rate (FDR) of 10%. With this threshold, a total of 160 (139 up-regulated and 21 down-regulated) genes were found to be differentially regulated. No differentially expressed genes were detected at FDR < 5%. Therefore, a less stringent 10% FDR cut-off was chosen for differential expression. No genes met the criteria for differential regulation using SAM with the same cut-off (FDR maximum of 10%) for the 0.5 and 2 mg/kg BW PCB 153 doses. Unsupervised hierarchical clustering analysis of the 160 differentially regulated genes was performed using Cluster3 software with average linkage distance metric [54]. Venn diagrams were drawn using an online tool Venny http://bioinfogp.cnb.csic.es/tools/venny/). The array data have been deposited in NCBI’s Gene Expression Omnibus (GEO) database (GEO accession GSE43733).

### Annotation and pathway analysis

For pathway analysis using the well annotated mammalian genome and proteome databases and tools, the Atlantic cod genes were mapped to human orthologs as described before [28]. Pathway enrichment analysis was performed in DAVID (Database for Annotation, Visualization and Integrated Discovery) [33] using Gene Ontology (GO), KEGG and Reactome databases. Enrichment analysis for GeneGo functional ontologies (Pathway Maps, Process Networks and GO processes), and analysis using Interactome and Network building tools was performed in MetaCore™ (GeneGo, St. Joseph, MI) [55]. The MetaCore default setting of false discovery rate (FDR) < 0.05 was used as threshold for significance in enrichment analysis.

### Gene Set Enrichment Analysis (GSEA)

Expression data for Atlantic cod genes that could be mapped to putative human orthologs (BLASTX with human UniProtKB/Swiss-Prot database, E-value < 10^−6^) (8.2 k genes in total) were used for gene set enrichment analysis (GSEA) [32]. Gene symbols for the 8.2 k unique genes were used as identifiers to perform GSEA. GSEA software and gene sets in the Molecular Signatures Database (MSigDB) [56] at Broad Institute (http://www.broadinstitute.org/gsea/index.jsp) were used. GSEA was performed with the control, 0.5, 2 and 8 mg/kg BW PCB 153 doses as continuous class labels with 1000 permutations of phenotypes. The absolute Pearson correlation metric was selected for ranking genes in descending order. The option for absolute correlation was chosen for ranking since it places the most differentially regulated (both up-regulated and down regulated) genes at the top of the ranked order and the least changing genes at the bottom. The curated Reactome, GenMAPP and KEGG gene sets in MSigDB were used. Gene sets enriched with FDR < 0.25 were considered significant as recommended [32].

### Quantitative real-time PCR (qPCR)

For each sample, cDNA synthesis was performed from total RNA (1.0 μg) using reverse- SuperScript III First-Strand Synthesis System for RT-PCR in 20 μL reaction as described in the manufacturer’s protocols (Invitrogen). qPCR assay and analysis was performed as described before [28]. Results are presented as means ± standard deviations. For confirmation of microarray results by qPCR, statistical analysis was performed on log_2_-transformed fold changes of expression (treated/control) using one-tailed Student’s t-test, and p < 0.05 was considered significant. For the qPCR assay of larger sample size, two samples (one from control and one from 2 mg/kg BW PCB 153 treated group) were found to be significant outliers (*p* < 0.05) using Grubb’s test in GraphPad outlier calculator (http://graphpad.com/quickcalcs/Grubbs1.cfm) and excluded from the analysis. For the qPCR assay of larger sample size, statistical analysis of log2-transformed fold-changes was performed using one-way ANOVA and Dunnett’s multiple comparison post-test (GraphPad Prism Software, La Jolla, CA, USA).

## Electronic supplementary material

Additional file 1: Table S1: The file contains 165 genes differentially regulated by PCB 153, which were used in pathway analysis. (XLS 52 KB)

Additional file 2: Figure S1: Comparison of fold changes of expression by microarray and qPCR; **Figure S2.** Enriched liver-specific ontologies; **Table S2.** A full list of significantly enriched GeneGo pathway maps; **Table S3.** Significantly enriched GO Processes; **Table S4.** significantly enriched top 20 GeneGo process networks; **Table S5.** significantly enriched Transcription Factors. (DOC 1 MB)

Additional file 3: **Is an excel document with a full list of significantly enriched Reactome gene sets.** (XLSX 13 KB)

## Abbreviations

PCB: Polychlorinated biphenyl
POP: Persistent organic pollutant
BW: Body weight
AhR: Aryl hydrocarbon receptor
SXR: Steroid and xenobiotic receptor
PXR: Pregnane X receptor
CAR: Constitutive androstane receptor
DAVID: Database for Annotation, Visualization and Integrated Discovery
SAM: Significance Analysis of Microarrays
FDR: False Discovery Rate
qPCR: Quantitative real-time polymerase chain reaction
GSEA: Gene Set Enrichment Analysis
FABP: Fatty acid binding protein
SREBP: Sterol regulatory element-binding protein
PPARG: Peroxisome proliferator-activated receptor gamma
PCNA: Proliferating Cell Nuclear Antigen
SCAP: SREBP chaperone
GO: Gene Ontology
BP: Biological process
ACLY: ATP citrate lyase
ACACA: Acetyl-CoA carboxylase (ACC)
ACSA: Acetyl-CoA synthetase
FASN: Fatty acid synthase
SCD5: Stearoyl-CoA desaturase 5
E2F1: EGR1, Early growth response 1
MAPK: Mitogen-activated protein kinase
KEGG: Kyoto Encyclopedia of Genes and Genomes
GenMAPP: Gene Map Annotator and Pathway Profiler
MCM5: Minichromosome Maintenance Complex Component 5
ES: Enrichment score
NES: Normalized ES
Nom p-val: Nominal p-value.
