# Supplementary material for: Liver transcriptome analysis of Atlantic cod (Gadus morhua) exposed to PCB 153 indicates effects on cell cycle regulation and lipid metabolism
Source: BMC Genomics. 2014 Jun 17;15(1):481. doi: 10.1186/1471-2164-15-481 (PMC4078240; doi:10.1186/1471-2164-15-481)
Supplement: Supplementary file 2 — Additional file 2: Figure S1: Comparison of fold changes of expression by microarray and qPCR; Figure S2. Enriched liver-specific ontologies; Table S2. A full list of significantly enriched GeneGo pathway maps; Table S3. Significantly enriched GO Processes; Table S4. significantly enriched top 20 GeneGo process networks; Table S5. significantly enriched Transcription Factors. (DOC 1 MB) [file 12864_2014_6174_MOESM2_ESM.doc]

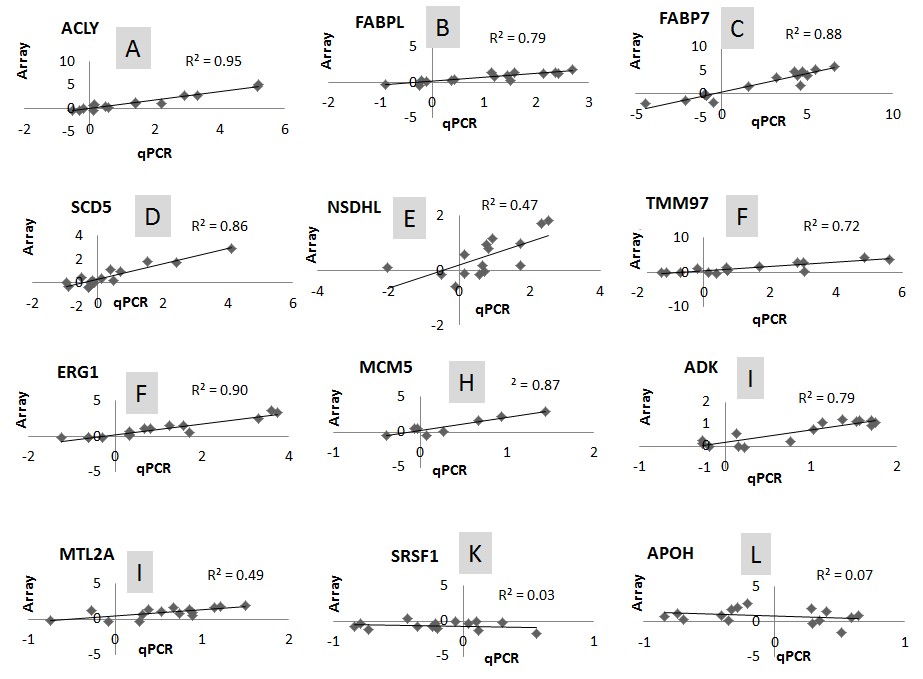


*

*

*

*

*

*

*

*

*

*

**Figure S1- Comparison of fold changes of expression by microarray and qPCR**.

qPCR was performed using same RNA samples as for microarray for control, 0.5 and 2 and 8 mg/kg BW PCB 153 doses (n = 3 for 2 mg/kg BW dose, and n = 4 for each of the other groups) . The star sign (*) shows significantly up-regulation (*p* < 0.05, one-tailed Student’s t-test) for the 8 mg/kg BW PCB 153 dose compared to control.

.


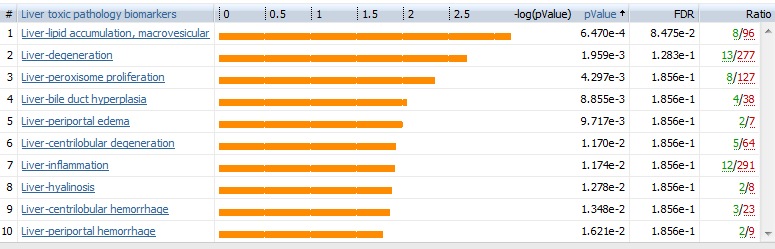


**A**


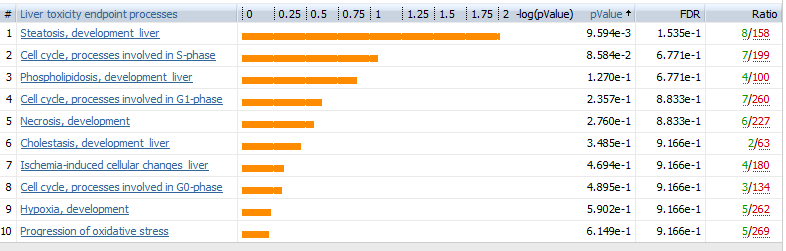


**B**


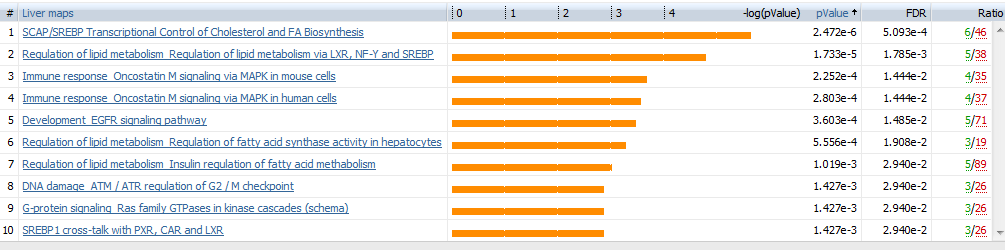


**C**


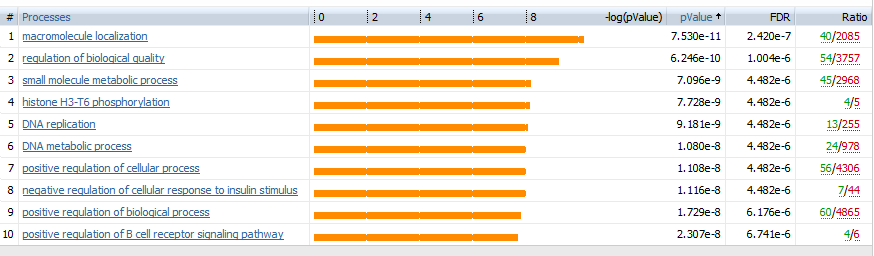


**D**


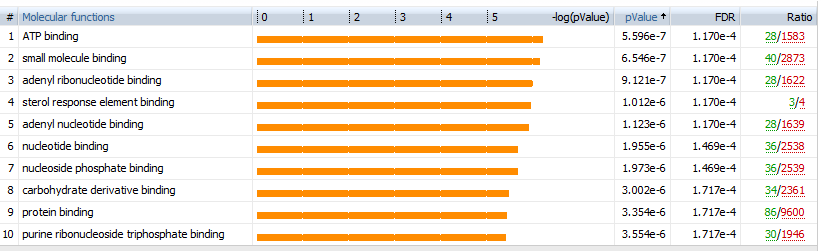


**E**


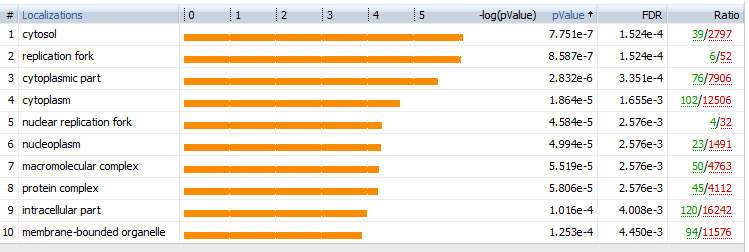


**F**


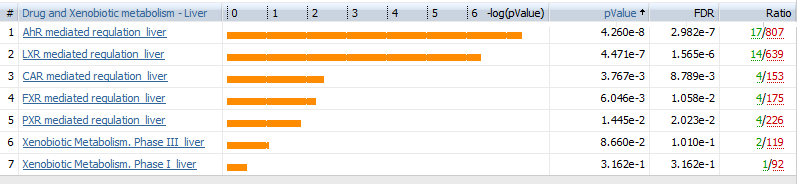


**G**


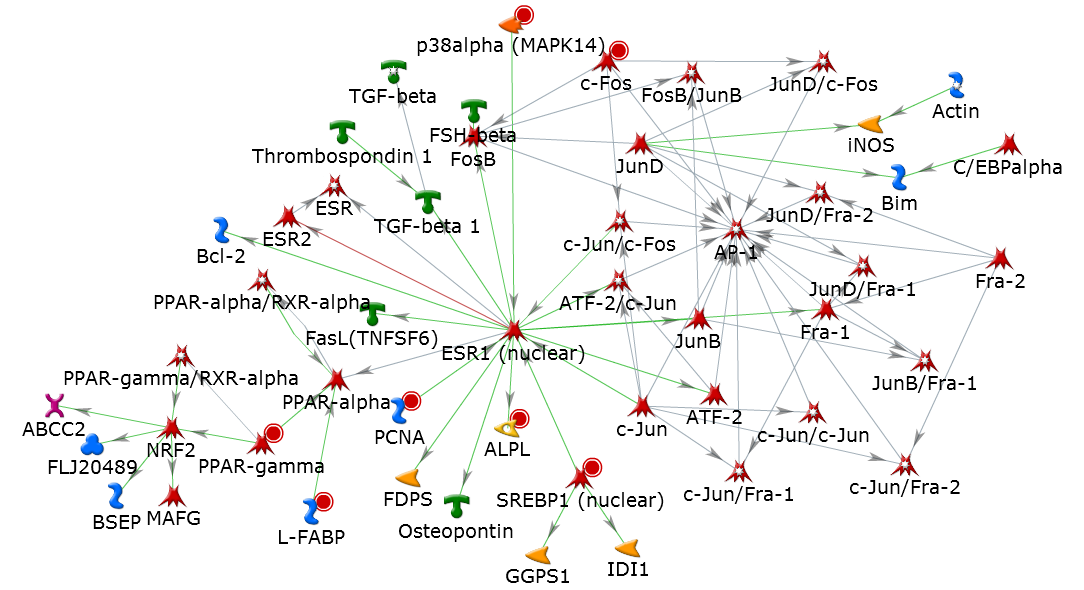


**H**


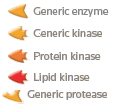

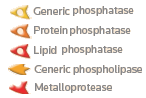

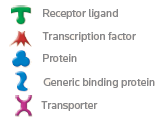

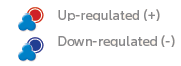

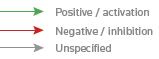


Legend

**Figure S2- Enriched liver-specific ontologies.**

Enriched liver-specific *Toxic Pathology Biomarkers* (**A**), *Toxicity Processes* (**B**), *Pathway* *Maps* (**C**), *GO Processes* (**D**), *GO Molecular Functions* (**E**), and *GO Localizations* (**F**), *Drug and Xenobiotic metabolism Enzymes* (**G**) and The most significant interaction network built from the genes in *Liver-lipid accumulation, macrovesicular* of the top enriched Toxic Pathology Biomarkers (**H**).

**Table S2. Significantly enriched GeneGo pathway maps**

| **#** | **Maps** | **p-value** | **FDR** | **Genes** |
| --- | --- | --- | --- | --- |
| 1 | SCAP/SREBP Transcriptional Control of Cholesterol and FA Biosynthesis | 6.0E-09 | 0.0 | HMDH, SREBP1 (Golgi membrane), ACLY, ACSA, SCD5, SREBP1 precursor, SREBP1 (nuclear), ACACA |
| 2 | Regulation of lipid metabolism_Regulation of lipid metabolism via LXR, NF-Y and SREBP | 2.1E-05 | 0.0 | SREBP1 (Golgi membrane), ACLY, SREBP1 precursor, SREBP1 (nuclear), ACACA |
| 3 | Adiponectin in pathogenesis of type 2 diabetes | 1.2E-04 | 0.0 | SREBP1 precursor, p38alpha (MAPK14), SREBP1 (nuclear), ACACA |
| 4 | Immune response_Oncostatin M signaling via MAPK in mouse cells | 2.6E-04 | 0.0 | EGR1, PPAR-gamma, p38 MAPK, c-Fos |
| 5 | Development_Role of IL-8 in angiogenesis | 2.8E-04 | 0.0 | HMDH, SREBP1 (Golgi membrane), SREBP1 precursor, SREBP1 (nuclear), c-Fos |
| 6 | Immune response_Oncostatin M signaling via MAPK in human cells | 3.2E-04 | 0.0 | EGR1, PPAR-gamma, p38 MAPK, c-Fos |
| 7 | Development_Gastrin in differentiation of the gastric mucosa | 3.5E-04 | 0.0 | PKC-beta, EGR1, PKC, cPKC (conventional) |
| 8 | Development_EGFR signaling pathway | 4.2E-04 | 0.0 | PKC-beta, p38 MAPK, p38alpha (MAPK14), c-Fos, Bax |
| 9 | Regulation of lipid metabolism_Regulation of fatty acid synthase activity in hepatocytes | 6.1E-04 | 0.0 | SREBP1 (Golgi membrane), SREBP1 precursor, SREBP1 (nuclear) |
| 10 | Regulation of lipid metabolism_Insulin regulation of fatty acid methabolism | 1.2E-03 | 0.0 | SREBP1 (Golgi membrane), ACLY, SREBP1 precursor, SREBP1 (nuclear), ACACA |
| 11 | SREBP1 cross-talk with PXR, CAR and LXR | 1.6E-03 | 0.0 | SREBP1 (Golgi membrane), SREBP1 precursor, SREBP1 (nuclear) |
| 12 | G-protein signaling_Ras family GTPases in kinase cascades (schema) | 1.6E-03 | 0.0 | p38 MAPK, p38alpha (MAPK14), c-Fos |
| 13 | DNA damage_ATM / ATR regulation of G2 / M checkpoint | 1.6E-03 | 0.0 | BLM, p38alpha (MAPK14), GADD45 beta |
| 14 | Cell cycle_Transition and termination of DNA replication | 2.0E-03 | 0.1 | PCNA, FEN1, POLD cat (p125) |
| 15 | Apoptosis and survival_p53-dependent apoptosis | 2.2E-03 | 0.1 | p38alpha (MAPK14), GADD45 beta, Bax |
| 16 | Renin-Angiotensin-Aldosterone System | 2.3E-03 | 0.1 | PKC-beta, CaMK I, p38alpha (MAPK14), c-Fos |
| 17 | Neuroprotective action of lithium | 2.4E-03 | 0.1 | p38 MAPK, p38alpha (MAPK14), Dsh, Bax |
| 18 | SREBP1 cross-talk with PXR, CAR and LXR/ Rodent version | 2.6E-03 | 0.1 | SREBP1 (Golgi membrane), SREBP1 precursor, SREBP1 (nuclear) |
| 19 | Development_Inhibition of angiogenesis by PEDF | 2.6E-03 | 0.1 | PPAR-gamma, p38 MAPK, Bax |
| 20 | DNA damage_ATM/ATR regulation of G1/S checkpoint | 2.9E-03 | 0.1 | PCNA, BLM, GADD45 beta |
| 21 | Apoptosis and survival_Role of CDK5 in neuronal death and survival | 3.4E-03 | 0.1 | EGR1, Bax, ErbB3 |
| 22 | Inhibition of neutrophil migration by proresolving lipid mediators in COPD | 3.5E-03 | 0.1 | p38 MAPK, PKC, Vinculin, VIL2 (ezrin) |
| 23 | Reproduction_GnRH signaling | 3.9E-03 | 0.1 | EGR1, CaMK I, p38alpha (MAPK14), c-Fos |
| 24 | HBV signaling via protein kinases leading to HCC | 4.0E-03 | 0.1 | PKC, cPKC (conventional), c-Fos |
| 25 | Immune response_IL-12-induced IFN-gamma production | 4.0E-03 | 0.1 | p38alpha (MAPK14), ERM, GADD45 beta |
| 26 | Cell adhesion_Role of tetraspanins in the integrin-mediated cell adhesion | 4.4E-03 | 0.1 | CD82, Vinculin, VIL2 (ezrin) |
| 27 | Regulation of metabolism_Bile acids regulation of glucose and lipid metabolism via FXR | 4.4E-03 | 0.1 | SREBP1 precursor, SREBP1 (nuclear), ACACA |
| 28 | Immune response_Role of integrins in NK cells cytotoxicity | 4.7E-03 | 0.1 | p38 MAPK, HLA-E, c-Fos |
| 29 | Development_PACAP signaling in neural cells | 5.1E-03 | 0.1 | cPKC (conventional), c-Fos, Bax |
| 30 | Development_BMP7 in brown adipocyte differentiation | 5.1E-03 | 0.1 | PPAR-gamma, BMP7, p38alpha (MAPK14) |
| 31 | Development_Growth hormone signaling via PI3K/AKT and MAPK cascades | 6.2E-03 | 0.1 | EGR1, p38alpha (MAPK14), c-Fos |
| 32 | Regulation of metabolism_Role of Adiponectin in regulation of metabolism | 6.7E-03 | 0.1 | SREBP1 precursor, p38alpha (MAPK14), ACACA |
| 33 | Development_VEGF signaling and activation | 6.7E-03 | 0.1 | PKC-beta, PKC, c-Fos |
| 34 | Development_VEGF signaling via VEGFR2 - generic cascades | 6.8E-03 | 0.1 | PKC-beta, p38 MAPK, PKC, Vinculin |
| 35 | Transport_Intracellular cholesterol transport | 7.1E-03 | 0.1 | MLN64, PLEKHA3 (FAPP1), Rab11-FIP2, Cyclophilin A |
| 36 | Signal transduction_Calcium signaling | 7.6E-03 | 0.1 | CaMK I, cPKC (conventional), VIL2 (ezrin) |
| 37 | Development_TGF-beta-dependent induction of EMT via MAPK | 8.5E-03 | 0.1 | p38 MAPK, p38alpha (MAPK14), c-Fos |
| 38 | Stimulation of TGF-beta signaling in lung cancer | 9.1E-03 | 0.1 | EGR1, p38alpha (MAPK14), Vinculin |
| 39 | Cytoskeleton remodeling_Integrin outside-in signaling | 9.6E-03 | 0.1 | Collagen II, Vinculin, Alpha-parvin |
| 40 | Regulation of lipid metabolism_Regulation of acetyl-CoA carboxylase 1 activity in lipogenic tissue | 9.9E-03 | 0.1 | SREBP1 (nuclear), ACACA |
| 41 | Neurophysiological process_Corticoliberin signaling via CRHR1 | 1.0E-02 | 0.1 | EGR1, cPKC (conventional), c-Fos |
| 42 | Development_GM-CSF signaling | 1.0E-02 | 0.1 | PKC-beta, EGR1, c-Fos |
| 43 | Immune response_C5a signaling | 1.0E-02 | 0.1 | p38 MAPK, PKC, c-Fos |
| 44 | Immune response_Function of MEF2 in T lymphocytes | 1.1E-02 | 0.1 | CaMK I, PKC, p38alpha (MAPK14) |
| 45 | Development_Differentiation of white adipocytes | 1.2E-02 | 0.1 | PPAR-gamma, p38alpha (MAPK14), SREBP1 (nuclear) |
| 46 | Immune response _IFN gamma signaling pathway | 1.2E-02 | 0.1 | p38 MAPK, PKR, MCM5 |
| 47 | Development_TGF-beta-induction of EMT via ROS | 1.4E-02 | 0.1 | p38 MAPK, p38alpha (MAPK14) |
| 48 | Immune response_CCR5 signaling in macrophages and T lymphocytes | 1.5E-02 | 0.1 | p38 MAPK, c-Fos, MIP-1-beta |
| 49 | Apoptosis and survival_NO signaling in apoptosis | 1.8E-02 | 0.1 | p38 MAPK, Bax |
| 50 | Development_Delta- and kappa-type opioid receptors signaling via beta-arrestin | 1.8E-02 | 0.1 | PKC, c-Fos |

**Table S3- Significantly enriched GO Processes as analyzed in MetaCore**

| **#** | **Processes** | **p-value** | **FDR** | **Count** |
| --- | --- | --- | --- | --- |
| 1 | macromolecule localization | 3.3E-11 | 1.1E-07 | 46 |
| 2 | small molecule metabolic process | 1.7E-09 | 2.8E-06 | 53 |
| 3 | single-organism metabolic process | 4.7E-09 | 5.3E-06 | 61 |
| 4 | regulation of biological quality | 6.6E-09 | 5.6E-06 | 60 |
| 5 | histone H3-T6 phosphorylation | 1.8E-08 | 1.2E-05 | 4 |
| 6 | protein localization | 2.7E-08 | 1.5E-05 | 37 |
| 7 | negative regulation of cellular response to insulin stimulus | 4.5E-08 | 2.0E-05 | 7 |
| 8 | positive regulation of B cell receptor signaling pathway | 5.2E-08 | 2.0E-05 | 4 |
| 9 | positive regulation of odontogenesis of dentin-containing tooth | 5.2E-08 | 2.0E-05 | 4 |
| 10 | response to external stimulus | 7.0E-08 | 2.4E-05 | 36 |
| 11 | DNA replication | 1.0E-07 | 3.2E-05 | 13 |
| 12 | negative regulation of transferase activity | 1.3E-07 | 3.7E-05 | 13 |
| 13 | histone-threonine phosphorylation | 2.4E-07 | 6.2E-05 | 4 |
| 14 | transport | 2.8E-07 | 6.6E-05 | 60 |
| 15 | lipoprotein transport | 3.8E-07 | 8.6E-05 | 6 |
| 16 | negative regulation of cellular process | 4.7E-07 | 9.8E-05 | 58 |
| 17 | establishment of localization | 4.9E-07 | 9.8E-05 | 60 |
| 18 | DNA strand elongation involved in DNA replication | 5.3E-07 | 1.0E-04 | 6 |
| 19 | DNA metabolic process | 5.8E-07 | 1.0E-04 | 24 |
| 20 | DNA strand elongation | 7.2E-07 | 1.2E-04 | 6 |
| 21 | regulation of cellular response to insulin stimulus | 8.0E-07 | 1.3E-04 | 7 |
| 22 | cellular lipid metabolic process | 8.3E-07 | 1.3E-04 | 25 |
| 23 | localization | 1.2E-06 | 1.8E-04 | 67 |
| 24 | negative regulation of catalytic activity | 1.3E-06 | 1.9E-04 | 22 |
| 25 | response to drug | 1.6E-06 | 2.2E-04 | 20 |
| 26 | regulation of B cell receptor signaling pathway | 1.7E-06 | 2.2E-04 | 4 |
| 27 | cellular metabolic process | 2.1E-06 | 2.5E-04 | 104 |
| 28 | negative regulation of cellular protein metabolic process | 2.1E-06 | 2.5E-04 | 18 |
| 29 | negative regulation of response to stimulus | 2.3E-06 | 2.6E-04 | 27 |
| 30 | positive regulation of cellular process | 2.4E-06 | 2.6E-04 | 59 |
| 31 | positive regulation of odontogenesis | 2.4E-06 | 2.6E-04 | 4 |
| 32 | lipid metabolic process | 2.9E-06 | 2.9E-04 | 28 |
| 33 | cellular nitrogen compound metabolic process | 2.9E-06 | 2.9E-04 | 72 |
| 34 | cellular component organization | 3.4E-06 | 3.4E-04 | 65 |
| 35 | negative regulation of protein metabolic process | 3.5E-06 | 3.4E-04 | 19 |
| 36 | regulation of catalytic activity | 3.8E-06 | 3.4E-04 | 38 |
| 37 | organic cyclic compound metabolic process | 3.8E-06 | 3.4E-04 | 72 |
| 38 | long-chain fatty acid transport | 3.8E-06 | 3.4E-04 | 6 |
| 39 | regulation of glucose transport | 4.0E-06 | 3.4E-04 | 8 |
| 40 | nervous system development | 4.2E-06 | 3.5E-04 | 41 |
| 41 | positive regulation of antigen receptor-mediated signaling pathway | 4.5E-06 | 3.5E-04 | 4 |
| 42 | skeletal muscle acetylcholine-gated channel clustering | 4.6E-06 | 3.5E-04 | 3 |
| 43 | protein import into nucleus, docking | 4.6E-06 | 3.5E-04 | 3 |
| 44 | negative regulation of biological process | 4.6E-06 | 3.5E-04 | 59 |
| 45 | negative regulation of protein modification process | 5.1E-06 | 3.8E-04 | 15 |
| 46 | heterocycle metabolic process | 5.5E-06 | 4.0E-04 | 69 |
| 47 | chemotaxis | 5.9E-06 | 4.0E-04 | 19 |
| 48 | dibenzo-p-dioxin metabolic process | 6.0E-06 | 4.0E-04 | 4 |
| 49 | positive regulation of biological process | 6.1E-06 | 4.0E-04 | 63 |
| 50 | metabolic process | 6.2E-06 | 4.0E-04 | 112 |

**Table S4. Significantly enriched top 20 GeneGo process networks**

| # | **Networks** | **p-value** | **FDR** | **Genes** |
| --- | --- | --- | --- | --- |
| 1 | Signal transduction_Leptin signaling | 1.3E-04 | 0.0 | SREBP1 (Golgi membrane), PPAR-gamma, EGR1, SREBP1 precursor, SREBP1 (nuclear), c-Fos, ACACA |
| 2 | Cytoskeleton_Actin filaments | 5.7E-04 | 0.0 | CIP4, ERM proteins, ARPC4, Alpha-fodrin, TRIPs, Myosin I, Vinculin, VIL2 (ezrin) |
| 3 | Signal transduction_WNT signaling | 6.0E-04 | 0.0 | PKC-beta, p38 MAPK, Casein kinase I, p38alpha (MAPK14), PKC-beta2, Casein kinase I epsilon, c-Fos, Dsh |
| 4 | Signal Transduction_Cholecystokinin signaling | 9.4E-04 | 0.0 | PKC-beta, p38 MAPK, CaMK I, p38alpha (MAPK14), PKC-beta2, c-Fos |
| 5 | Development_Neuromuscular junction | 9.8E-04 | 0.0 | PKC-beta, DVL-1, PKC-beta2, Vinculin, cPKC (conventional), Dsh, ErbB3 |
| 6 | Cell cycle_S phase | 1.1E-03 | 0.0 | MCM3, PCNA, FEN1, POLD cat (p125), POLA2, MCM5, GADD45 beta |
| 7 | Immune response_Phagocytosis | 2.6E-03 | 0.0 | PKC-beta, ERM proteins, p38 MAPK, Myosin I, p38alpha (MAPK14), Vinculin, c-Fos, VIL2 (ezrin) |
| 8 | Signal Transduction_BMP and GDF signaling | 2.9E-03 | 0.0 | PPAR-gamma, p38 MAPK, BMP7, Collagen II, GADD45 beta |
| 9 | Immune response_Phagosome in antigen presentation | 4.4E-03 | 0.1 | ERM proteins, p38 MAPK, PSMD3, PSMA5, PSMB7, p38alpha (MAPK14), Vinculin, VIL2 (ezrin) |
| 10 | Reproduction_Male sex differentiation | 4.8E-03 | 0.1 | HMDH, PKC-beta, p38 MAPK, PKC, BMP7, PKR, p38alpha (MAPK14), Bax |
| 11 | Reproduction_Gonadotropin regulation | 5.4E-03 | 0.1 | PKC-beta, EGR1, p38 MAPK, CaMK I, p38alpha (MAPK14), PKC-beta2, c-Fos |
| 12 | Regulation of metabolism_Bile acid regulation of lipid metabolism and negative FXR-dependent regulation of bile acids concentration | 7.0E-03 | 0.1 | SREBP1 (Golgi membrane), SREBP1 precursor, SREBP1 (nuclear), ACACA |
| 13 | Cell adhesion_Integrin-mediated cell-matrix adhesion | 8.0E-03 | 0.1 | ERM proteins, CD82, Lpd, Collagen II, Vinculin, Alpha-parvin, VIL2 (ezrin) |
| 14 | Signal transduction_ESR1-nuclear pathway | 8.4E-03 | 0.1 | PPAR-gamma, DSCAM, SREBP1 precursor, p38alpha (MAPK14), SREBP1 (nuclear), c-Fos, ErbB3 |
| 15 | Reproduction_GnRH signaling pathway | 8.8E-03 | 0.1 | PKC-beta, EGR1, p38 MAPK, CaMK I, p38alpha (MAPK14), PKC-beta2 |
| 16 | Neurophysiological process_Circadian rhythm | 8.9E-03 | 0.1 | EGR1, Casein kinase I epsilon, cPKC (conventional), c-Fos |
| 17 | DNA damage_Checkpoint | 1.1E-02 | 0.1 | p38 MAPK, PCNA, BLM, p38alpha (MAPK14), GADD45 beta |
| 18 | Apoptosis_Anti-Apoptosis mediated by external signals via MAPK and JAK/STAT | 1.2E-02 | 0.1 | EGR1, p38 MAPK, PKR, cPKC (conventional), c-Fos, Bax |
| 19 | Cell adhesion_Cadherins | 1.3E-02 | 0.1 | PKC-beta, PKC, Casein kinase I, Casein kinase I epsilon, Vinculin, Dsh |
| 20 | Signal transduction_NOTCH signaling | 1.3E-02 | 0.1 | p38 MAPK, PCNA, DVL-1, p38alpha (MAPK14), c-Fos, Dsh, ErbB3 |

**Table S5. Significantly enriched Transcription Factors (FDR < 0.05)^a^**

| **IDs in active data set** | **Object name** | **Actual** | **n** | **R** | **N** | **Expected** | **Ratio** | **z-score** | **p-value** |
| --- | --- | --- | --- | --- | --- | --- | --- | --- | --- |
|  | NP220 | 2 | 165 | 2 | 24963 | 1.3E-02 | 151 | 17.3 | 4.3E-05 |
|  | ZNF16 | 1 | 165 | 1 | 24963 | 6.6E-03 | 151 | 12.3 | 6.6E-03 |
|  | SPZ1 | 1 | 165 | 1 | 24963 | 6.6E-03 | 151 | 12.3 | 6.6E-03 |
|  | OBP2A | 1 | 165 | 1 | 24963 | 6.6E-03 | 151 | 12.3 | 6.6E-03 |
|  | MSK1 | 1 | 165 | 1 | 24963 | 6.6E-03 | 151 | 12.3 | 6.6E-03 |
|  | FBXL10 | 1 | 165 | 1 | 24963 | 6.6E-03 | 151 | 12.3 | 6.6E-03 |
|  | TAX1BP1 | 1 | 165 | 1 | 24963 | 6.6E-03 | 151 | 12.3 | 6.6E-03 |
|  | hnRNP-G | 1 | 165 | 1 | 24963 | 6.6E-03 | 151 | 12.3 | 6.6E-03 |
|  | BAF60c | 1 | 165 | 1 | 24963 | 6.6E-03 | 151 | 12.3 | 6.6E-03 |
|  | DMBX1 | 1 | 165 | 1 | 24963 | 6.6E-03 | 151 | 12.3 | 6.6E-03 |
|  | HINT | 1 | 165 | 1 | 24963 | 6.6E-03 | 151 | 12.3 | 6.6E-03 |
|  | NAB2 | 1 | 165 | 1 | 24963 | 6.6E-03 | 151 | 12.3 | 6.6E-03 |
|  | SOHLH1 | 1 | 165 | 1 | 24963 | 6.6E-03 | 151 | 12.3 | 6.6E-03 |
|  | UEV1A | 1 | 165 | 1 | 24963 | 6.6E-03 | 151 | 12.3 | 6.6E-03 |
|  | FLJ20449 | 1 | 165 | 1 | 24963 | 6.6E-03 | 151 | 12.3 | 6.6E-03 |
|  | HUC | 1 | 165 | 1 | 24963 | 6.6E-03 | 151 | 12.3 | 6.6E-03 |
|  | FOXI2 | 1 | 165 | 1 | 24963 | 6.6E-03 | 151 | 12.3 | 6.6E-03 |
|  | CREB5 | 1 | 165 | 1 | 24963 | 6.6E-03 | 151 | 12.3 | 6.6E-03 |
|  | NXF | 2 | 165 | 7 | 24963 | 4.6E-02 | 43 | 9.1 | 8.9E-04 |
|  | Elk-3 | 2 | 165 | 14 | 24963 | 9.3E-02 | 22 | 6.3 | 3.8E-03 |
|  | CIC | 2 | 165 | 17 | 24963 | 1.1E-01 | 18 | 5.7 | 5.5E-03 |
|  | AP-2B | 3 | 165 | 26 | 24963 | 1.7E-01 | 17 | 6.8 | 6.6E-04 |
|  | RARbeta | 3 | 165 | 32 | 24963 | 2.1E-01 | 14 | 6.1 | 1.2E-03 |
|  | SREBP2 (nuclear) | 6 | 165 | 66 | 24963 | 4.4E-01 | 14 | 8.5 | 5.0E-06 |
|  | DLX5 | 2 | 165 | 23 | 24963 | 1.5E-01 | 13 | 4.8 | 1.0E-02 |
|  | CDX1 | 3 | 165 | 37 | 24963 | 2.4E-01 | 12 | 5.6 | 1.9E-03 |
|  | FOXD3 | 2 | 165 | 25 | 24963 | 1.7E-01 | 12 | 4.5 | 1.2E-02 |
|  | GLI-3 | 2 | 165 | 25 | 24963 | 1.7E-01 | 12 | 4.5 | 1.2E-02 |
|  | ATF-1 | 4 | 165 | 62 | 24963 | 4.1E-01 | 9.8 | 5.6 | 7.6E-04 |
| P36956 | SREBP1 (nuclear) | 8 | 165 | 140 | 24963 | 9.3E-01 | 8.6 | 7.4 | 4.4E-06 |
|  | SNAIL1 | 4 | 165 | 71 | 24963 | 4.7E-01 | 8.5 | 5.2 | 1.3E-03 |
|  | E2F2 | 6 | 165 | 108 | 24963 | 7.1E-01 | 8.4 | 6.3 | 8.4E-05 |
|  | EGR2 (Krox20) | 4 | 165 | 72 | 24963 | 4.8E-01 | 8.4 | 5.1 | 1.3E-03 |
|  | C/EBP zeta | 5 | 165 | 91 | 24963 | 6.0E-01 | 8.3 | 5.7 | 3.5E-04 |
|  | E2F6 | 4 | 165 | 78 | 24963 | 5.2E-01 | 7.8 | 4.9 | 1.8E-03 |
|  | COUP-TFII | 4 | 165 | 85 | 24963 | 5.6E-01 | 7.1 | 4.6 | 2.5E-03 |
|  | ATF-3 | 4 | 165 | 87 | 24963 | 5.8E-01 | 7.0 | 4.5 | 2.7E-03 |
|  | WT1 | 6 | 165 | 136 | 24963 | 9.0E-01 | 6.7 | 5.4 | 2.9E-04 |
|  | PBX1 | 4 | 165 | 92 | 24963 | 6.1E-01 | 6.6 | 4.4 | 3.3E-03 |
|  | NFYB | 5 | 165 | 116 | 24963 | 7.7E-01 | 6.5 | 4.9 | 1.1E-03 |
|  | DEC1 (Stra13) | 3 | 165 | 70 | 24963 | 4.6E-01 | 6.5 | 3.7 | 1.1E-02 |
|  | E2F4 | 13 | 165 | 306 | 24963 | 2.0E+00 | 6.4 | 7.8 | 1.4E-07 |
|  | NFYA | 5 | 165 | 121 | 24963 | 8.0E-01 | 6.3 | 4.7 | 1.3E-03 |
|  | E2A | 4 | 165 | 97 | 24963 | 6.4E-01 | 6.2 | 4.2 | 4.0E-03 |
|  | MITF | 5 | 165 | 129 | 24963 | 8.5E-01 | 5.9 | 4.5 | 1.7E-03 |
|  | RBP-J kappa (CBF1) | 4 | 165 | 108 | 24963 | 7.1E-01 | 5.6 | 3.9 | 5.8E-03 |
|  | STAT1 | 11 | 165 | 319 | 24963 | 2.1E+00 | 5.2 | 6.2 | 9.6E-06 |
|  | E2F3 | 5 | 165 | 146 | 24963 | 9.7E-01 | 5.2 | 4.1 | 2.9E-03 |
|  | ATF-2 | 5 | 165 | 148 | 24963 | 9.8E-01 | 5.1 | 4.1 | 3.1E-03 |
|  | EBF | 4 | 165 | 121 | 24963 | 8.0E-01 | 5.0 | 3.6 | 8.6E-03 |
|  | E2F1 | 24 | 165 | 746 | 24963 | 4.9E+00 | 4.9 | 8.7 | 1.6E-10 |
|  | C/EBPalpha | 10 | 165 | 331 | 24963 | 2.2E+00 | 4.6 | 5.3 | 7.5E-05 |
|  | PPAR-alpha | 5 | 165 | 166 | 24963 | 1.1E+00 | 4.6 | 3.8 | 5.0E-03 |
|  | HNF3-alpha | 5 | 165 | 166 | 24963 | 1.1E+00 | 4.6 | 3.8 | 5.0E-03 |
|  | USF1 | 6 | 165 | 210 | 24963 | 1.4E+00 | 4.3 | 3.9 | 2.8E-03 |
|  | IRF8 | 6 | 165 | 218 | 24963 | 1.4E+00 | 4.2 | 3.8 | 3.4E-03 |
|  | SMAD3 | 5 | 165 | 193 | 24963 | 1.3E+00 | 3.9 | 3.3 | 9.3E-03 |
|  | IRF4 | 11 | 165 | 433 | 24963 | 2.9E+00 | 3.8 | 4.9 | 1.5E-04 |
| P18146 | EGR1 | 10 | 165 | 410 | 24963 | 2.7E+00 | 3.7 | 4.5 | 4.2E-04 |
|  | Androgen receptor | 20 | 165 | 856 | 24963 | 5.7E+00 | 3.5 | 6.2 | 1.0E-06 |
|  | AP-2A | 6 | 165 | 259 | 24963 | 1.7E+00 | 3.5 | 3.3 | 7.7E-03 |
|  | NANOG | 15 | 165 | 700 | 24963 | 4.6E+00 | 3.2 | 4.9 | 6.7E-05 |
|  | SP3 | 10 | 165 | 474 | 24963 | 3.1E+00 | 3.2 | 3.9 | 1.3E-03 |
|  | GATA-1 | 7 | 165 | 352 | 24963 | 2.3E+00 | 3.0 | 3.1 | 9.1E-03 |
|  | p63 | 8 | 165 | 409 | 24963 | 2.7E+00 | 3.0 | 3.3 | 6.0E-03 |
|  | SP1 | 28 | 165 | 1490 | 24963 | 9.8E+00 | 2.8 | 6.0 | 5.2E-07 |
|  | HNF4-alpha | 8 | 165 | 440 | 24963 | 2.9E+00 | 2.8 | 3.0 | 9.1E-03 |
|  | c-Myc | 41 | 165 | 2435 | 24963 | 1.6E+01 | 2.5 | 6.6 | 1.5E-08 |
|  | ESR1 (nuclear) | 19 | 165 | 1163 | 24963 | 7.7E+00 | 2.5 | 4.2 | 2.6E-04 |
|  | c-Jun | 11 | 165 | 712 | 24963 | 4.7E+00 | 2.3 | 3.0 | 7.9E-03 |
|  | Oct-3/4 | 18 | 165 | 1182 | 24963 | 7.8E+00 | 2.3 | 3.7 | 8.6E-04 |
|  | p53 | 14 | 165 | 966 | 24963 | 6.4E+00 | 2.2 | 3.1 | 5.0E-03 |
|  | GCR-alpha | 15 | 165 | 1173 | 24963 | 7.8E+00 | 1.9 | 2.7 | 1.1E-02 |
|  | CUX1 (p110) | 18 | 165 | 1420 | 24963 | 9.4E+00 | 1.9 | 2.9 | 6.1E-03 |
|  | CREB1 | 54 | 165 | 5150 | 24963 | 3.4E+01 | 1.6 | 3.9 | 1.8E-04 |

| **^a^ Columns have the following meaning:** | |
| --- | --- |
| **IDs in active data set** | original probe/gene IDs in the activated dataset(s) |
| **Object id** | a network object id corresponding to the given gene |
| **Object name** | network object name in MetaBase |
| **Actual** | number of targets in the activated dataset(s) regulated by the chosen transcription factor (TF) |
| **n** | number of network objects in the activated dataset(s) |
| **R** | number of targets in the complete database or background list regulated by the chosen TF |
| **N** | total number of gene-based objects in the complete database or background list |
| **Expected** | mean value for hypergeometric distribution (n*R/N) |
| **Ratio** | connectivity ratio (Actual/Expected) |
| **z-score** | z-score ((Actual-Expected)/sqrt(variance)) |
| **p-value** | probability to have the given value of Actual or higher (or lower for negative z-score) |
